# Depression, anxiety, and history of substance abuse among Norwegian inmates in preventive detention: Reasons to worry?

**DOI:** 10.1186/1471-244X-11-40

**Published:** 2011-03-10

**Authors:** Henning Værøy

**Affiliations:** 1 Dept. of Psychiatric R & D, Akershus University Hospital, 1478 Lørenskog, Norway

## Abstract

**Background:**

Inmates on preventive detention are a small and select group sentenced to an indefinite term of imprisonment. Mood disorders and substance abuse are risk factors for inmate violence and recidivism, so the prevalence of depression, anxiety, and substance abuse was examined in this cohort using psychometric tests.

**Methods:**

Completion of self-report questionnaires was followed by face-to-face clinical interviews with 26 of the 56 male inmates on preventive detention in Norway's Ila Prison. Substance abuse histories and information about the type of psychiatric treatment received were compiled. To assess anxiety and depression, the Hospital Anxiety and Depression Scale (HADS), the Clinical Anxiety Scale (CAS), and the Montgomery Asberg Depression Rating Scale (MADRS) were used.

**Results:**

Scores on the MADRS revealed that 46.1% of inmates had symptoms of mild depression. The HADS depression subscale showed that 19.2% scored above the cut-off for depression (κ = 0.57). The CAS anxiety score was above the cut-off for 30.7% of the subjects, while 34.6% also scored above the cut-off on the HADS anxiety subscale (κ = 0.61). Almost 70% of all these inmates, and more than 80% of those convicted of sex crimes, had a history of alcohol and/or drug abuse.

**Conclusions:**

Mild anxiety and depression was found frequently among inmates on preventive detention. Likewise, the majority of the inmates had a history of alcohol and drug abuse. Mood disorders and substance abuse may enhance recidivism, so rehabilitation programs should be tailored to address these problems.

## Background

In Norway, inmates on preventive detention are a group of offenders at high risk for recommitting violent crimes. The options available to Norwegian courts of law are either a time limited or a time unlimited (indefinite) term. The latter sentence is relatively rare and is applied only in cases in which the perpetrators have committed serious violent crimes. A sentence to preventive detention also indicates that the courts consider the defendant at high risk for reoffending, and thus an imminent threat to society. There are two forms of time unlimited sentences. Offenders judged legally insane are sentenced to indefinite psychiatric treatment, while those considered mentally fit are sentenced to preventive detention. The law states that prisoners on time unlimited sentences should have their cases reconsidered after some time served. Following a new hearing, the courts then decide whether or not the time unlimited sentence should be continued.

According to Norwegian law, only perpetrators that are liable to be sentenced to psychiatric treatment must undergo a forensic psychiatric examination. For this reason, a prisoner sentenced to an indefinite term may have an unknown or incompletely determined mental health status.

The number of these preventive detention sentences is small, so these inmates represent a select group within the Norwegian prison population. According to recent Norwegian prison statistics, there were only 61 such prisoners nationwide as of January 2008 [1]. Some studies have addressed mental health issues in Norwegian inmates [2-5], but none have examined these indefinite-term prisoners using face-to-face interviews. In one study, Kjelsberg et al. [2] studied the mental health status of this prison population (61 inmates) but assessments were based on information from the prisoners' files from the prison health department. Screening for psychiatric symptoms was, according to Kjelsberg et al. [2], performed during the inmates' first week of imprisonment by prison staff without formal mental health training. From these data, Kjelsberg et al. [2] estimated that 25% of inmates showed signs of clinical anxiety and 38% showed signs of depression.

The present study was performed approximately two years after the study by Kjelsberg et al. [2]. The same prison population on preventive detention was examined in both studies; however, some of the inmates may have been released and replaced by others during the two year period. Regrettably, no information is available about the degree of overlap between participating inmates in the two studies. The findings reported by Kjelsberg et al. [2] are of special interest because they provide the first estimate of the rates of anxiety and depression in detainees on preventive detention.

A recent study on the same population of detainees found that 73% acknowledged a risk for reoffending if released, and 69% were critical of the rehabilitation offered and saw no crime-preventive effect [6]. A link between aggressive behaviour and affective disorders has been established by several studies. Some have shown that negative affect increased the likelihood of impulsive antisocial or aggressive behaviour [7] and that affective factors are involved in non-completion of treatment for high-risk offenders [8]. On the other hand, it has also been reported that the use of antidepressants may reduce impulsive violent behaviour [9].

According to the Office for National Statistics Prison Survey in England and Wales from 1997 [10], the prevalence of alcohol and substance abuse in male prisoners is 63% for alcohol abuse and 66% for drug abuse. In a review by Fazel et al. [11], data from seven surveys of alcohol abuse/dependence among 4141 male prisoners revealed that the prevalence ranged from 17.7% to 30.0%, while the rate of drug abuse/dependence ranged from 10% to 48%. Indeed, alcoholism has been found to be more than twice as common among prisoners than in the general population and drug-dependence eight times as common [11].

There are few studies available describing the present Norwegian group of inmates on preventive detention. Based on the literature, there is reason to suspect a high prevalence of psychiatric disorders (anxiety, depression, and substance abuse) in our population, but the extent is uncertain. Greater knowledge of the mental health status of prisoners under preventive detention may provide useful information for developing crime prevention measures and for successful rehabilitation programs.

Apart from information released by the criminal justice system and the prison through publicly available statistics, information from the prisoners' personal health files was not made available for this study. Information provided by the participants has therefore not been controlled against the information registered in the inmates' personal health files. All data presented in this report are based on the inmates' own statements and results from psychometric tests.

## Methods

This study was performed at Ila prison, a national high security facility in a suburb of Oslo. The Ila prison is the main prison in Norway for male convicts on preventive detention. The prison has 12 departments, of which 4 house inmates on preventive detention. At all times, a total of around 120 prisoners are serving time, limited and unlimited, in this facility.

Once the study was approved by prison authorities and the Regional Committee for Research Ethics (Approval no. 601-07195a 1.2007.1723), the perpetrators were asked to participate. The participants were initially presented written information regarding the study. This was distributed by prison guards during the winter of 2008-2009. As part of the recruitment process, two psychiatrists visited the different departments at the prison in order to talk directly to the inmates, provide extra information where needed, and to answer any questions regarding the study.

Those who agreed to participate signed the required consent form. Appointments were then booked at the prison hospitality area on a random basis according to each prisoner's daily program and available time. The inmates' personal data were anonymized.

### Participants

The inmates examined were all males on preventive detention. This indefinite sentence also implies that the court has determined that the accused suffered from no psychiatric conditions requiring sentence to treatment. Since January 2005, a total of 105 men have been sentenced to preventive detention in Norway. Thirty-four have been released following appeal, 7 are serving time elsewhere, and 3 are dead. Of the 105 inmates at Ila, 78.1% are registered as having one or more psychiatric diagnoses, 77.1% as having addiction problems, and 9.5% as previously psychotic [1]. The total number of inmates in preventive detention by January 2008 was 61 [1]. The Norwegian Bureau of Statistics (Statistics Norway, 2009 update) reported 3412 prisoners in 2007, an incarceration rate of 91 inmates per 100,000 inhabitants [12].

Those who agreed to participate in the present study (28/56) represented 50% of the total number of inmates on preventive detention at the time [6]. Two foreign citizens were excluded due to language problems; thus 26 inmates were interviewed for this study (participation rate of 46.4%).

No information was available about the non-participants apart from their sentence and their male gender. The mean age of the participants was 42.5 years (range, 24-55 years) and all were born Norwegians. The median length of sentence was 8 years (range, 3 to 21 years). Of the 26 inmates, 6 underwent a forensic psychiatric examination before being sentenced and 20 did not. At the time of imprisonment, 5 inmates were married or living in a stable relationship, and 4 had children. Twenty inmates were single, of which 10 were fathers. Fifteen detainees reported no psychiatric complaints at the time of the interview, 7 reported problems with both anxiety and depression, 2 reported depressive symptoms, 1 reported anxiety, and 1 reported recent brief psychotic episodes.

### Psychometric tests

In order to screen for symptoms of anxiety and depression, the inmates were given the Hospital Anxiety and Depression Scale (HADS) [13] at the beginning of the interview. The HADS inventory was chosen primarily because of its screening power and the fact that it was not developed for a mentally disordered population. Furthermore, it takes only between 2-5 minutes to complete.

During the clinical interview that followed, the Clinical Anxiety Scale (CAS) [14] and the Montgomery Asberg Depression Rating Scale (MADRS) [15] were applied in order to ascertain a test-based diagnostic prevalence of anxiety and depression.

By choosing the CAS, it was possible to measure the current level of anxiety in each individual. The CAS was also chosen because its brevity makes it easy to administer. Furthermore, the CAS complements the MADRS, as it shows little overlap on items for anxiety in the latter [16]. The MADRS was chosen based on research demonstrating that MADRS in tandem with the CAS provides a robust measure of the level of depression which can be graded according to the scores obtained [16]. It is also easy to administer. In 1985, the HAD subscales were validated against both the MADRS and the CAS [17]. Thus, the tests used in this study are well validated against each other.

The HADS self-assessment scale is only valid for screening purposes [18]. An evaluation of the severity of anxiety and depression based on the 7 item HADS subscales [18] suggested that a score of 0-7 for either subscale should be regarded as within the normal range, while a score of 11 or higher indicates the probable presence of a mood disorder. A score of 8-10 is only considered suggestive of a mood disorder. A cut-off score of 8 was chosen for both HADS subscales in this study [13]. It is also believed that applying the same cut-off used in other studies provides a common reference level for comparison. Likewise, 8 was the cut-off for the CAS, although two subgroups were noted, one in which the CAS score was between 8 and 13 (mild anxiety) and one in which the score was ≥ 14 [14].

In this study, the definition of mild, medium, and severe depression on the MADRS scale were taken from Snaith et al. [16] who graded the scores of the MADRS and the CAS scales. The severity of depression was measured with the MADRS [15] and was classified as follows: no depression (< 7), mild depression [7-19], moderate depression [20-34], and severe depression (35-60) [16].

According to the authors [16], the differences between grade score ranges were significant for both the CAS and the MADRS scales. The levels of statistical significance were at least p < 0.05, indicating that the scale scores allow valid comparisons [17].

Table 1 displays the number of inmates testing at or above the respective cut-off point values for the different scales applied.

**Table 1:** Male inmates on preventive detention: Test scores and self-reported treatments

Nr	Self-reported current psychotropic medication	Self-reported current psychological treatment	Self-reported group treatment	HADS	CAS	HADS	MADRS
*				Anxiety subscale Cut-off point ≥ 8	Cut-off point ≥ 8	depression subscale Cut-off point ≥ 8	Cut-off point ≥ 7
1	Yes	Yes	No				7

2		Yes	No	9	8		

3		No	No	13		10	10

4		Yes	No	12			13

5		Yes	No		12	12	16

6		No	No		10		10

7		Yes	Yes	8	9		

8		Yes	No		9		9

9	Yes	Yes	No	8			8

10	Yes	Yes	No	9	8		11

11	Yes	Yes	No	16	9	12	13

12		Yes	No	14		15	12

13		No	No				12

14	Yes	No	No	13	18	8	31

*Inmates with scores below the respective cut-off points are not included in the table.

### Alcohol and drug abuse

As part of the investigation, the inmates were specifically asked about their use of alcohol and drugs (substance abuse) prior to arrest and incarceration. The number of substance abusing prisoners is expected to be high. Specific questions regarding substance abuse were asked, including *"Prior to your arrest and incarceration, did you use alcohol and/or drugs on a regular basis? If yes, which was your substance of first choice?"*. The inmates' answers to these questions were recorded. Likewise, the detainees were asked about the age of first use and the substance taken.

### Treatment

During the interview sessions, the inmates were asked whether they received any kind of treatment. If the inmates acknowledged that they had received treatment, they were then asked to explain, to the best of their ability, what kind of treatment they received. The prisoners' answers varied in exactness and details, but could be clustered in three groups: pharmacological treatment, individual treatment by a psychologist, and group treatment. This data is presented in table 1. Apart from the inmates confirming that they received treatment and their own descriptions of this treatment, no other information as to the kind of medication prescribed or the content of the group-based and individually based therapy was made available. Table 1 also displays the individual therapeutic measures as reported by each prisoner. The choice to display this in table 1 is based on the assumption that ongoing treatment may have influenced the grades of depression and anxiety presented.

Occasionally the prisoners could be specific as to which drug, dosages, and duration of drug use; however, in most cases they did not remember sufficient details in order for the investigator to systemize these data for deeper analysis.

### Statistics

Given the low number of available participants, the use of statistics was limited to calculating *kappa *values. All other data is presented as percentages without error estimates.

## Results

At the time of the investigation, all subjects had either participated in rehabilitation programs or were currently participating in one. The test scores for anxiety and depression for inmates on preventive detention are shown in table 1. Only 5 of the inmates were receiving some form of psychotropic medication at the time of the study. Eleven inmates were receiving psychological treatment, either as part of group therapy program (one inmate), individual treatment, or both (10 inmates).

The MADRS scale revealed that 12 of the 26 inmates (46.1%) scored positively for mild depression. In contrast, results from the HADS depression subscale indicated that only 5 of the 26 (19.2%) scored positively (*Kappa *= 0.57). For anxiety, 34.6% of the detainees scored positively on the HADS anxiety subscale and 30.7% scored positively on the CAS (*Kappa *= 0.61). All the CAS scores, except one, were indicative of mild anxiety symptoms (scores between 8-13 points). The only exception scored 18 points, despite receiving psychotropic medication. The same prisoner also scored higher on the MADRS scale.

Based on the inmates' self-reported history, 69.3% had a history of alcohol and/or drug abuse and 42.3% reported first use before the age of 13. Alcohol was the most frequently used debut substance. Table 2 displays the various subgroups within the cohort of inmates based on the crimes committed. In the group who had committed sexually related crimes, almost 82% had a history of substance abuse. Eleven prisoners had committed sexual offences as their main crime. Among the eight prisoners who had been sentenced for attempted homicide or homicide, five (62.5%) also reported a history of alcohol and drug abuse.

**Table 2:** Criminal offence and history of alcohol and drug abuse before sentencing among twenty-six inmates on preventive detention.

Offence	Number of inmates with a reported history	Number of inmates with no reported history	Percentage in crime category reporting substance abuse
Homicide/Attempted	5/8		62.5%

Sexually related offence	9/11		81.8%

Other violence	4/6		66.7%

No history of alcohol and drug abuse	-	8	-

## Discussion

Inmates sentenced to preventive detention are amongst the most violent in the Norwegian prison system. This study documents the self-reported history of substance abuse and mental health status based on multiple psychometric tests. Affective conditions are associated with an enhanced risk for prison violence and recidivism [9], while alcoholism is frequently found among prisoners [11]. This study confirms relatively high rates of mood disorders and alcoholism/drug-dependence in this population, underscoring the need for better rehabilitation strategies that include treatments for these psychiatric disorders.

The present results found a slight increase in symptoms compared to Kjelsberg et al. [2] who found that 25% of the inmates had clinical anxiety and 38% had some degree of clinical depression. However, there was no information given on how the diagnoses were made or the severity of symptoms [2]. Furthermore, the data obtained by Kjelsberg et al. [2] were collected during the inmates' first week of imprisonment by prison employees not formally trained in mental health care [2]. In the present study, we used face-to-face interviews and several well-validated tests for depression and anxiety. In addition, the length of time each prisoner had served was different, and this factor may have contributed to the differences observed between the two studies. Other factors that may have contributed to the current prevalence of mild depression and anxiety, apart from the obvious impact of imprisonment itself, e.g caffeine and smoking, is a matter of speculation. The scant previous literature gives contradictory results both regarding depression and the potential influence of caffeine intake [19,20] and for depression and the possible negative effects of smoking [21-23].

Both pharmaceutical compounds and drugs of abuse may induce depression and anxiety as side effects. During the interviews with the inmates, no access to information regarding their use of medications was given. Thus, the reported use of drugs as "psychotropic medication" in this study (table 1) is entirely based on self-report. Some inmates could not remember the name, the dosage, or the treatment duration. In most cases however, they knew why the drug had been prescribed. The prisoners were also asked if they used legal drugs inappropriately and whether they used illegal substances. This was denied by all except for two prisoners who confirmed that they had tried illegal drugs for the first time during their current imprisonment. Thus, there is reason to suspect that illegal drugs are available within the high security facility.

Mental disorders and substance abuse are risk factors for increased criminal recidivism, particularly for violent and sexual offences [24,25]. Few studies have examined temperament and character traits as possible predictors for anxiety and depression. A study addressing this issue [24] found a relationship between temperament, depression, and anxiety using the Temperament Character Inventory (TCI) [26]. The TCI was also found useful in identifying prisoners with a history of substance abuse [24]. Prisoners who had injected drugs during the past 12 months scored higher on the Novelty Seeking (NS) and Harm Avoidance (HA) scales, and lower on Persistence (PS), Self-directedness (SD), and Cooperativeness (CO) scales than non-injectors [24]. Studies using Cloninger's character traits [27,28] have found a positive correlation between the severity of substance abuse and character traits manifesting immaturity, self-destructiveness, irresponsibility, and an inability to define or pursue meaningful goals (SD). Likewise, inmates with a severe substance abuse problem were less likely to be cooperative, empathic, compassionate, helpful, or to focus on the needs of others (CO). In contrast, the more severe the substance use, the more likely the inmates were to show a greater intrinsic desire for self-actualization, creativity, and spirituality (ST) [26,27].

In Ila prison's rehabilitation programs, the inmates are offered mental health services either in the form of group therapy or as individual consultations. A study [6] that focused on the inmates' expectations of gain from the different programs provided by the criminal justice system found that 58% had negative experiences. The main criticism was that the programs were not sufficiently targeted towards each individual's needs and that the prison staff leading the various program sessions did not possess the necessary competence. Therefore, it is possible that some inmates, given their lack of thrust in the services offered [6], underreported their symptoms.

Table 2 shows the distribution of substance abuse in subgroups of inmates based on the offences committed. The number of inmates on preventive detention with a history of substance abuse (18/26) was approximately 70%, consistent with previous studies showing that the effects of alcohol are significantly associated with incarceration for violent crime [29]. There is also evidence linking alcohol use or abuse with aggressive sex crimes, that the role of alcohol consumption seems to be greater in sexual offenders targeting boys than in those targeting girls, and that substance abuse contributed to recidivism [30]. A study from New Zealand [31] found that 81% of male prisoners in a medium/minimum security prison had some kind of lifetime DSM III alcohol disorder, while 30% had a severe lifetime drug disorder.

In another study, Fazel et al. [11] concluded that the prevalence of substance abuse and dependence among prisoners was much higher in prisoners than in the general population. Among the studies confirming a link between substance abuse and sex offenders, one study found that 85% of 113 convicted male sexual offenders had a lifetime diagnosis of substance abuse disorder according to the DSM IV criteria [32]. Another study [33] found that sex offenders have significant difficulties with alcohol abuse.

In agreement with others [25,30], in the present study a history of substance abuse was most frequently reported by those inmates who had committed sexual offences (81.8%). Of those prisoners convicted of a non-sexual violent crime (64.3%) had a confirmed history of substance abuse. In light of these data, it is evident that rehabilitation of prisoners should focus largely on substance abuse issues. In addition, when dealing with subgroups of inmates classified as having several specific risk factors for reoffending, rehabilitation must encompass these accordingly.

The low number of participants in this study was due to the relatively small population of detainees on preventive detention in Norway. This has obvious consequences since a low participation rate limits the strength of our conclusions. At the time of the investigation, 56 of 61 inmates on preventive detention were available at the prison and the current response rate was 28 of 56 (50%). While this response rate is low compared to many convenience sampling studies, this is a unique population with few similar studies from which to draw comparisons. However, when the present study was presented to the prison administration, it was underlined that according to their experience, a participation rate of around 25-30% might be obtainable, but rarely more. It is not known if the investigators' visits to the different departments to recruit study subjects influenced the response rate.

As for the non-participating prisoners in preventive detention, we have little information. During the interviews, however, some of the inmates commented that there were non-participants in great need of psychiatric assistance in their department. Consent to use information from the prisoners' files was not given by the ethics committee without each prisoner's written consent. Asking for the inmates' consent to study their personal files most likely would have reduced the participation rate even further. This assumption is based on previous observations and experience with prisoners, and their expressed suspicion towards the prison and the criminal justice system. Questions asked by the inmates during the recruitment process underscored the inmates' suspicions. Their main concerns were whether or not the information given would be forwarded to the prison administration or otherwise made accessible to anyone who could use the information against them. Thus, it was decided not to ask for the inmates' permission to access personal files.

## Conclusions

The present findings show a relatively high prevalence of mild anxiety and depression among inmates on preventive detention. In light of the established link between mood disorders and violence, these findings are cause for concern. Indeed, it has been shown that symptoms of depression, regardless of whether a specific disorder diagnosis has been made, were associated with multiple incidents of fighting [34].

The environment in the prison may play a role in the development of both anxiety and depressive symptoms, as can factors like smoking/cessation and illegal use of drugs within the prison facilities. Anyhow, symptoms of anxiety and depression concomitant with a history of alcohol and drug abuse are significant risk factors for violence.

The prevalence of alcohol and drug abuse was high, especially among those who had committed sexually related crimes. A relationship between substance abuse and sex offences necessitates preventive measures that focus on substance abuse in addition to aberrant sexual behaviours. Successful rehabilitation programs may reduce these risk factors, thereby enhancing prison safety and reducing recidivism.

## Competing interests

The authors declare that they have no competing interests.

## Authors' contributions

The author is the main investigator.

## Pre-publication history

The pre-publication history for this paper can be accessed here:

http://www.biomedcentral.com/1471-244X/11/40/prepub
